# Phospholipid Phosphatase 4 promotes proliferation and tumorigenesis, and activates Ca^2+^-permeable Cationic Channel in lung carcinoma cells

**DOI:** 10.1186/s12943-017-0717-5

**Published:** 2017-08-29

**Authors:** Xin Zhang, Lan Zhang, Bihua Lin, Xingxing Chai, Ronggang Li, Yuehua Liao, Xinghui Deng, Qiongru Liu, Wenli Yang, Yubo Cai, Wei Zhou, Zhichao Lin, Wenhai Huang, Meigong Zhong, Fangyong Lei, Jinhua Wu, Shuaishuai Yu, Xiaoping Li, Shangren Li, Yueyue Li, Jincheng Zeng, Wansheng Long, Dong Ren, Yanming Huang

**Affiliations:** 1 Clinical Experimental Center, Jiangmen Central Hospital, Affiliated Jiangmen Hospital of Sun Yat-sen University, Jiangmen, 529030 China; 2 Department of Pathology, Jiangmen Central Hospital, Affiliated Jiangmen Hospital of Sun Yat-sen University, Jiangmen, 529030 China; 3 Dongguan Key Laboratory of Medical Bioactive Molecular Developmental and Translational Research, Guangdong Provincial Key Laboratory of Medical Molecular Diagnostics, Guangdong Medical University, Dongguan, 523808 China; 4 Guangdong Provincial Key Laboratory of Malignant Tumor Epigenetics and Gene Regulation, Department of Obstetrics and Gynecology, Sun Yat-Sen Memorial Hospital, Sun Yat-sen University, Guangzhou, 510120 China; 5 Laboratory Animal Center, Guangdong Medical University, Zhanjiang, 524023 China; 6 Department of Thoracic Surgery, Jiangmen Central Hospital, Affiliated Jiangmen Hospital of Sun Yat-sen University, Jiangmen, 529030 China; 7 Department of Pharmacy, Jiangmen Maternity and Child Health Care Hospital, Jiangmen, 529030 China; 8 Department of Oncology, Jiangmen Central Hospital, Affiliated Jiangmen Hospital of Sun Yat-sen University, Jiangmen, 529030 China; 9 Department of Clinical Laboratory, Jiangmen Central Hospital, Affiliated Jiangmen Hospital of Sun Yat-sen University, Jiangmen, 529030 China; 10 Department of General Surgery, Jiangmen Central Hospital, Affiliated Jiangmen Hospital of Sun Yat-sen University, Jiangmen, 529030 China; 11 Department of Radiology, Jiangmen Central Hospital, Affiliated Jiangmen Hospital of Sun Yat-sen University, Jiangmen, 529030 China; 12 Department of Orthopaedic Surgery, The First Affiliated Hospital of Sun Yat-sen University, 58# Zhongshan 2rd Road, Guangzhou, Guangdong Province 510080 China; 13 Department of Respiration Medicine, Jiangmen Central Hospital, Affiliated Jiangmen Hospital of Sun Yat-sen University, Jiangmen, 529030 China

**Keywords:** PLPP4, Proliferation, Cell cycle, Tumorigenesis, Ca^2+^-permeable cationic channel, Therapeutic target, Lung carcinoma

## Abstract

**Background:**

Phospholipid phosphatase 4 (PPAPDC1A or PLPP4) has been demonstrated to be involved in the malignant process of many cancers. The purpose of this study was to investigate the clinical significance and biological roles of PLPP4 in lung carcinoma.

**Methods:**

PLPP4 expression was examined in 8 paired lung carcinoma tissues by real-time PCR and in 265 lung carcinoma tissues by immunohistochemistry (IHC). Statistical analysis was performed to evaluate the clinical correlation between PLPP4 expression and clinicopathological features and survival in lung carcinoma patients. In vitro and in vivo assays were performed to assess the biological roles of PLPP4 in lung carcinoma. Fluorescence-activated cell sorting, Western blotting and luciferase assays were used to identify the underlying pathway through which PLPP4 silencing mediates biological roles in lung carcinoma.

**Results:**

PLPP4 is differentially elevated in lung adenocarcinoma (ADC) and lung squamous cell carcinoma (SQC) tissues. Statistical analysis demonstrated that high expression of PLPP4 significantly and positively correlated with clinicopathological features, including pathological grade, T category and stage, and poor overall and progression-free survival in lung carcinoma patients. Silencing PLPP4 inhibits proliferation and cell cycle progression in vitro and tumorigenesis in vivo in lung carcinoma cells. Our results further reveal that PLPP4 silencing inhibits Ca^2+^-permeable cationic channel, suggesting that downregulation of PLPP4 inhibits proliferation and tumorigenesis in lung carcinoma cells via reducing the influx of intracellular Ca^2+^.

**Conclusion:**

Our results indicate that PLPP4 may hold promise as a novel marker for the diagnosis of lung carcinoma and as a potential therapeutic target to facilitate the development of novel treatment for lung carcinoma.

**Electronic supplementary material:**

The online version of this article (10.1186/s12943-017-0717-5) contains supplementary material, which is available to authorized users.

## Background

Lung carcinoma is the leading cause of cancer-related deaths worldwide [1]. Although great progress since the introduction of third-generation anti-neoplastic agents and individualized targeted therapy, such as epidermal growth factor receptor (EGFR) tyrosine kinase inhibitors, the overall 5-year survival rate is still modest [2]. Therefore, an in-depth understanding of potential biomarkers and the therapeutic targets of lung carcinoma will facilitate to the development of novel therapeutic strategy.

Lipid phosphate phosphatases (LPPs), also known as phospholipid phosphatase (PLPP), belong to a superfamily of integral membrane glycoproteins and have six putative transmembrane domains and three highly conserved domains. The conserved domains are paralleled with the transmembrane domains where they form the active sites of the phosphatases [3, 4]. LPPs exert multifarious functions via catalyzing the dephosphorylation of a number of bioactive lipid mediators including phosphatidic acid (PA), lysophosphatidic acid (LPA) and sphingosine 1-phosphate (S1P), to attenuate cell activation. Moreover, LPPs can also generate alternative signals with additional biological activities from phosphatidate, sphingosine and ceramide [3]. Among their versatile biological roles, the fact that LPPs can convert the phosphatidate generated from phosphatidylcholine within the cell by phospholipase D to diacylglycerol is worth noting. This increase in diacylglycerol concentration has been referred to as the second phase of diacylglycerol signaling in which LPPs are believed to play an important role, which further results in the release and influx of intracellular Ca^2+^ [5].Furthermore, accumulating evidence has shown that aberrant expression of LPPs has been implicated in the development and progression of cancer. For example, through analyzing a comparative genomic hybridization array and expression profiling data for a series of 152 ductal breast cancer tissues and 21 breast cancer cell lines, Bernard-Pierrot and colleagues reported that PLPP5 was overexpressed, which contributed to cell survival and cell transformation. Importantly, the oncogenic properties of PLPP5 were further demonstrated by its ability to transform NIH-3 T3 fibroblasts, further inducing their anchorage-independent growth [6]; in oral squamous cell carcinoma (OSCC) patients, PLPP3 was downregulated in approximately 70% OSCC patients and PLPP3 expression negatively correlated with TNM stage and tumor volume [7]. Moreover, Mahmood et al. reported that overexpression of PLPP5 caused by amplification of the 8p11-12 chromosomal region, a common genetic event in many epithelial cancers, was found in several cancers, including breast cancer, pancreatic adenocarcinomas and lung carcinoma [8]. Thus, these studies indicated that different LPPs exert oncogenic or tumor-suppressive functions depending on the tumor types.

In this study, by analyzing the expression levels of LPP family proteins in lung carcinoma RNA expression profile datasets from The Cancer Genome Atlas (TCGA), we found that PLPP4 is dramatically elevated compared with other LPPs in lung carcinoma tissues. Our results further verify that overexpression of PLPP4 is observed in lung carcinoma tissues and cells and positively correlates with advanced clinicopathological features and poor prognosis in lung carcinoma patients. Silencing PLPP4 inhibits the proliferation and tumorigenicity of lung carcinoma cells both in vitro and in vivo. In addition, our findings reveal that downregulating PLPP4 inhibits Ca^2+^-permeable cationic channel in lung carcinoma cells. Taken together, our findings indicate that PLPP4 plays an important role in the progression of lung carcinoma and suggest that PLPP4 may serve as a potential target for human lung carcinoma treatment.

## Methods

### Cell culture

The human lung carcinoma cell lines A549, Calu-3, NCI-H226, NCI-H292, NCI-H358, NCI-H1650 and NCI-H1975, and a normal lung epithelial cell line WI-38 were obtained from the Shanghai Chinese Academy of Sciences Cell Bank (China). All cells were cultured in RPMI-1640 medium (Life Technologies, Carlsbad, CA, US) supplemented with penicillin G (100 U/ml), streptomycin (100 mg/ml) and 10% fetal bovine serum (FBS, Life Technologies) and were grown under a humidified atmosphere of 5% CO2 at 37 °C7.

### Patients and tumor tissues

Five paired lung adenocarcinoma tissues, 2 paired lung squamous cell carcinoma tissues and 1 lung adenosquamous carcinoma tissue and the 8 corresponding matched adjacent tumor normal tissues, and 43 tissues from benign pulmonary lesions were obtained during surgery, and the clinicopathological features of the patients are summarized in Additional file 1: Table S1 and Additional file 2: Table S2. The total 265 paraffin-embedded, archived non-small cell lung cancer samples were obtained from surgery or needle biopsy, and the clinicopathological features of the patients are summarized in Additional file 3: Table S3. All tissues were collected from the Clinical Biobank of Collaborative Innovation Center for Medical Molecular Diagnostics of Guangdong Province, The Affiliated Jiangmen Hospital of Sun Yat-sen University (Guangdong, China) between January 2016 and December 2016.Patients were diagnosed based on clinical and pathological evidence, and the specimens were immediately snap-frozen and stored in liquid nitrogen tanks. For the use of these clinical materials for research purposes, prior patients’ consents and approval from the Institutional Research Ethics Committee were obtained. The proportions of tumor vs. non-tumor in H&E-stained tissue samples were evaluated by two independent professional pathologists. The tumor proportions in all clinical lung cancer tissue samples analyzed in this study exceeded 75%.

### RNA extraction, reverse transcription, and real-time PCR

Total RNA from tissues or cells was extracted using the RNA Isolation Kit-miRNeasy Mini Kit (Qiagen, USA) according to the manufacturer’s instructions. Messenger RNA (mRNA) was reverse transcribed from the total mRNA using the Revert Aid First Strand cDNA Synthesis Kit (Thermo, USA) according to the manufacturer’s protocol. Complementary DNA (cDNA) was amplified and quantified on a CFX96 system (BIORAD, USA) using iQ SYBR Green (BIO-RAD, USA). The primers are provided in Additional file 4: Table S4. Real-time PCR was performed according to a standard method, as previously described [9]. Primers for glyceraldehyde-3-phosphate dehydrogenase (GAPDH) were synthesized and purified by RiboBio (Guangzhou, China). GAPDH was used as an endogenous control for mRNA. Relative fold expressions was calculated using the comparative threshold cycle (2^-ΔΔCt^) method.

### Vectors and retroviral infection

Short hairpin (shRNA) RNA for human PLPP4 was cloned into a phU6-MCS-Ubiquitin -EGFP-IRES-puromycin plasmid (GV280, Genechem, China), and the list of primers for the clone reactions is presented in Additional file 5: Table S5. Transfection of the plasmids was performed using Lipofectamine 3000 reagent (Invitrogen, USA) according to the manufacturer’s instructions. Stable cell lines expressing shPLPP4#1or shPLPP4#2 were generated by lentiviral infection using HEK293T cells, and selected with 0.5 mg/L puromycin for 10 days. The luciferase reporter system of pE2F-luc, pRb-luc and pGL3-luc (Clontech) was used to examine the transcriptional activity of E2F and Rb.

### Cell counting kit-8 analysis and colony formation assay

For cell counting kit-8 analysis, cells (2 × 10^3^) were seeded into 96 well plates and the specific staining process and methods were performed according to the previous study [10]. For colony formation assay, cells (0.2 × 10^3^) were plated into six well plates and cultured for 10 days. Colonies were then fixed for 15 min with 10% formaldehyde and stained for 30s with 1.0% crystal violet. Plating efficiency was calculated as previously described [11]. Different colony morphologies were captured under a light microscope (Olympus).

### Anchorage-independent growth ability assay

Cells (3 × 10^3^) were suspended in 2 ml of complete medium plus 0.3% agar (Sigma-Aldrich, St Louis, MO, US). The agar-cell mixture was plated as a top layer onto a bottom layer of 0.6% complete medium agar mixture as previously described [12]. After 14 days of culture, the colony size was measured using an ocular micrometer and colonies >0.1 mm in diameter were counted.

### Cell cycle analysis

Pretreatment and staining were performed using the Cell Cycle Detection Kit (KeyGEN, China) according to the manufacturer’s instructions. Cells (5 × 10^5^) were harvested by trypsinization, washed with ice-cold phosphate-buffered saline (PBS) and fixed in 75% ice-cold ethanol in PBS. Before staining, cells were gently resuspended in cold PBS, and ribonuclease was added to the cells’ suspension tube, and incubated at 37 °C for 30 min, followed by incubation with propidium iodide(PI) for 20 min at room temperature. Cell samples (2 × 10^4^) were then analyzed by FACSCanto II flow cytometer (Becton, Dickinson and Company, Franklin Lakes, NJ, US) and the data were analyzed using FlowJo 7.6 software (TreeStar Inc., Ashland, OR, US).

### High throughput data processing and visualization

The RNA sequencing profiles of lung adenocarcinoma and lung squamous cell carcinoma were downloaded from The Cancer Genome Atlas (TCGA; https://cancergenome.nih.gov/) and analyzed using Excel 2010 and GraphPad 5 software. The 17 RNA expression profiles of non-small cell lung cancer based on Affymetrix U133 Plus2.0 microarray were downloaded from ArrayExpress (http://www.ebi.ac.uk/arrayexpress/). Integrated analysis of all data collected from TCGA and ArrayExpress using the YuGene program is summarized in Additional file 6: Table S6 [13], including 340 normal lung tissues and 2032 lung cancer tissues. The integrative expression profile of lung cancer was named for the AE-meta dataset.

### Immunohistochemistry

The immunohistochemistry procedure and scoring of PLPP4 expression were performed as previously described [14]. The slides were incubated overnight at 4 °C in a humidified chamber with anti-PLPP4 antibodies (Novus Biologicals: NBP2-14545) diluted 1:100 in PBS. Scores given by the two independent investigators were averaged for further comparative evaluation of PLPP4 expression. Tumor cell proportion were scored as follows: 0 (no positive tumor cells); 1 (<10% positive tumor cells); 2 (10–35% positive tumor cells); 3 (35–70% positive tumor cells) and 4 (>70% positive tumor cells). Staining intensity was graded according to the following criteria: 0 (no staining); 1 (weak staining, light yellow); 2 (moderate staining, yellow brown) and 3 (strong staining, brown). The staining index (SI) was calculated as the product of the staining intensity score and the proportion of positive tumor cells. Based on this method of assessment, PLPP4 expression in lung carcinoma samples was evaluated by the SI, with scores of 0, 1, 2, 3, 4, 6, 8, 9 or 12. A SI score of 4 was the median for all sample tissues SI socres. High and low expression of PLPP4 were stratified according to the following criteria: SI ≤ 4 was used to define tumors with low expression of PLPP4, and SI score 6 was used to define tumors with high expression of PLPP4.

### Dual luciferase report experiments

Cells (5 × 10^5^) were plated in 60-mm cell culture dishes, and allowed to proliferate to 60–80% confluence after 24 h of culture, and the reporter constructs were transfected into cells using Lipofectamine 3000. After a 12-h incubation, the transfection medium was replaced; cells were harvested and washed with PBS, and lysed with passive lysis buffer (Promega). The cell lysates were analyzed immediately using the Synergy™ 2 microplate system (BioTek, Winooski, VT, US). Luciferase and *Renilla* luciferase were measured using a Dual-Luciferase Reporter Assay System (Promega) according to the manufacturer’s instructions. The luciferase activity of each lysate was normalized to the *Renilla* luciferase activity. The relative transcriptional activity was converted to the fold induction above the vehicle control value.

### Western blotting

Nuclear/cytoplasmic fractions were separated by using the Cell Fractionation Kit (Cell Signaling Technology, USA) according to the manufacturer’s instructions, and whole cell lysates were extracted using RIPA Buffer (Cell Signaling Technology). Western blots were performed according to a standard method, as previously described [15]. Antibodies against cyclin D1, cyclin A2 and cyclin B1 were purchased from Cell Signaling Technology (Cyclin Antibody Sampler Kit: Cat#9869) (Danvers, MA, USA), and PLPP4 (Cat#: ab150925), NFAT1 (Cat#: ab49161), p-NFAT1 (Cat#: ab200819) and p84 (Cat#: ab102684) from Abcam. The membranes were stripped and reprobed with an anti–α-tubulin antibody (Cell Signaling Technology. Cat#: 2125) as the loading control.

### Statistical analysis

All values are presented as the mean ± standard deviation (SD). Significant differences were determined using GraphPad 5.0 software (USA). Student’s t-test was used to determine significant differences between two groups. One-way ANOVA was used to determine statistical differences between multiple groups. The chi-square test was used to analyze the relationship between PLPP4 expression and clinicopathological characteristics. Survival curves were plotted using the Kaplan-Meier method and compared by log-rank test. *P* < 0.05 was considered significant. All the experiments were repeated three times.

## Results

### PLPP4 is upregulated in lung carcinoma tissues and cell lines

**Fig. 1:**
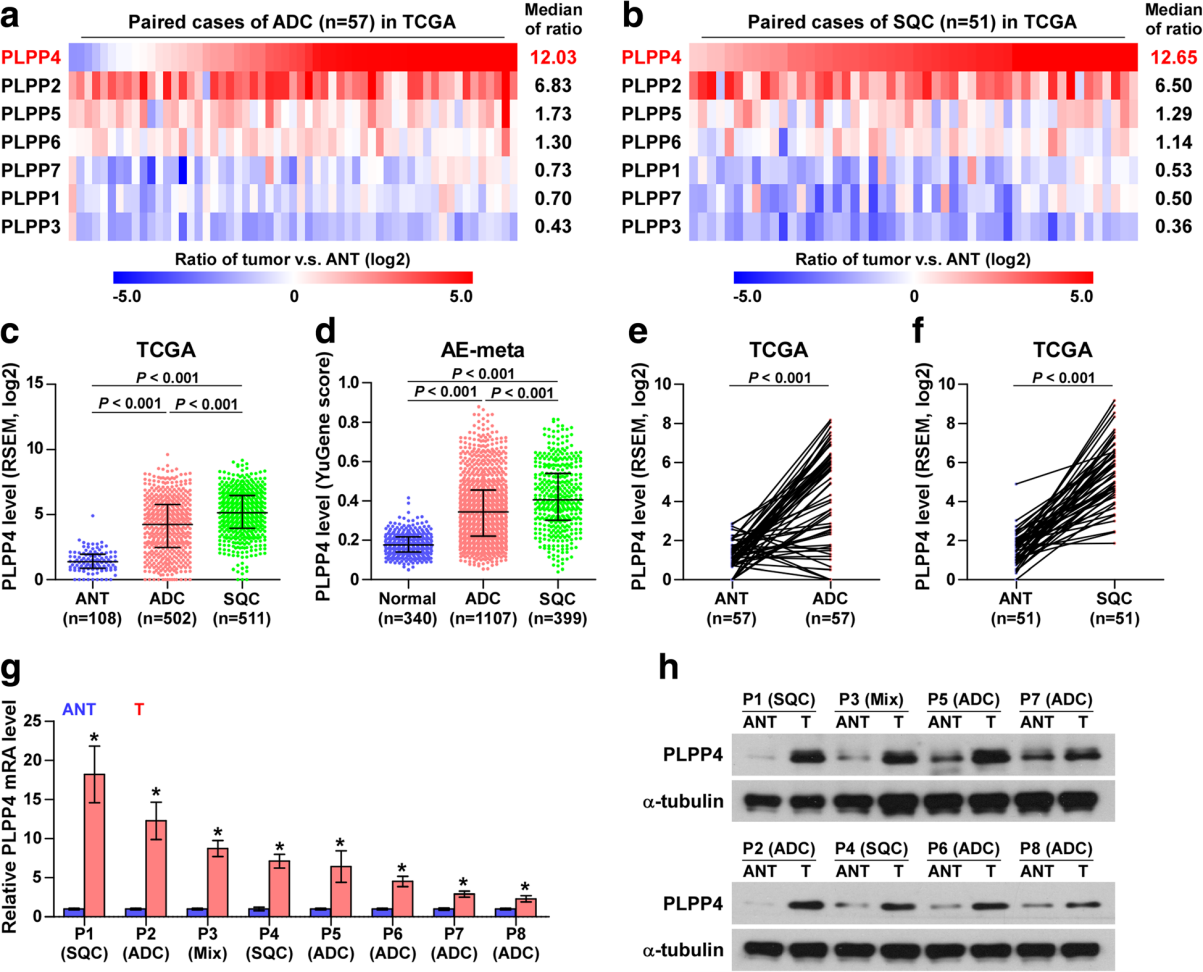
PLPP4 is upregulated in lung carcinoma. **a** PLPP1-7 expression levels in the RNA sequencing profiles of 57 paired lung adenocarcinoma (ADC) tissues from TCGA dataset. **b** PLPP1-7 expression levels in the RNA sequencing profile of 51 paired lung squamous cell carcinoma (SQC) tissues from TCGA dataset. **c** Expression of PLPP4 in the adjacent normal tissues (ANT), ADC tissues and SQC tissues in TCGA lung carcinoma profiles. **d** Expression of PLPP4 in the adjacent normal tissues (ANT), ADC tissues and SQC tissues in the lung carcinoma profiles from ArrayExpress. **e** PLPP4 expression was upregulated in the RNA sequencing profile of 57 ADC tissues from TCGA dataset compared with that in ANT. **f** PLPP4 expression was upregulated in the RNA sequencing profile of 51 SQC tissues from TCGA dataset compared with that in ANT. **g** and **h** mRNA and protein expression levels of PLPP4 in eight paired lung tissues, including 5 paired ADC tissues, 2 paired SQC tissues and 1 lung adenosquamous carcinoma tissues (Mix). α-Tubulin was used as a loading control. The average PLPP4 mRNA expression level was normalized to the expression of GAPDH. Each bar represents the mean values ± SD of three independent experiments. *P < 0.05

We first analyzed the expression levels of LPPs proteins, including PLPP1-7, in the high throughput paired lung carcinoma RNA expression profile datasets from TCGA and found that the expression levels of PLPP2, PLPP4, PLPP5 and PLPP6 were differentially elevated in lung adenocarcinoma (ADC) and lung squamous cell carcinoma (SQC) tissues compared to the respective adjacent normal tissues (ANT), particularly PLPP4 with a 12.03-fold change in ADC and a 12.65-fold change in SQC (Fig. 1a and b). Conversely, the expression levels of PLPP1, PLPP3 and PLPP7 were decreased to varying degrees compared with those in the ANT (Fig. 1a and b), indicating that different members of the PLPP family have oncogenic or tumor-suppressive roles in the development of lung carcinoma. Subsequent analysis of PLPP4 expression in lung carcinoma datasets from TCGA and ArrayExpress showed that PLPP4 expression was upregulated in ADC tissues compared that in the ANT and was further increased in SQC tissues (Fig. 1c and d). PLPP4 expression levels in 57 paired ADC tissues and 51 paired SQC tissues from lung carcinoma TCGA profile were analyzed and the results revealed that PLPP4 expression was dramatically elevated in both ADC and SQC tissues compared with that in the respective ANT (Fig. 1e and f). We further examined PLPP4 expression in our 5 paired ADC tissues, 2 paired SQC tissues and 1 lung adenosquamous carcinoma tissue (Mix: P3) and found that the mRNA and protein expression levels of PLPP4 were differentially elevated in tumor tissues compared with those in the respective ANT (Fig. 1g and h). Therefore, our results indicated that PLPP4 may be implicated in the development and progression of lung carcinoma.

### High expression of PLPP4 correlates with advanced clinicopathological features in lung carcinoma patients

**Fig. 2:**
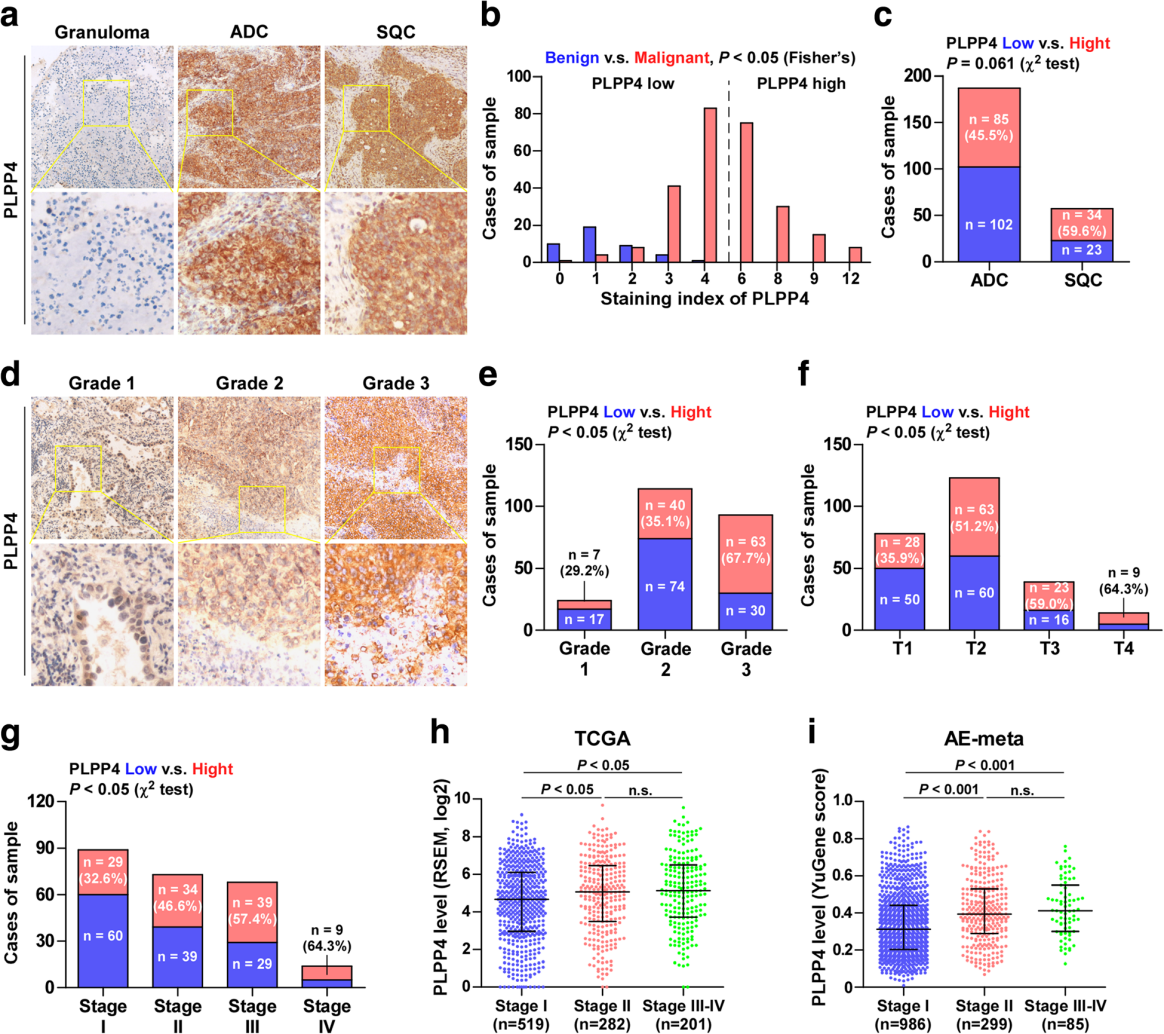
High PLPP4 expression correlates with advanced clinicopathological features in lung carcinoma patients. **a** Representative images of PLPP4 expression in lung granuloma tissues, ADC tissues and SQC tissues. **b** The number of lung granuloma tissues and lung carcinoma tissues stratified by IHC staining index. **c** Percentages and number of samples showing high or low PLPP4 expression in ADC and SQC tissues. **d** Representative images of PLPP4 expression in lung carcinoma tissues with different clinical grade. **e** Percentages and number of samples showing high or low PLPP4 expression in lung carcinoma tissues with different grades. **f** Percentages and number of samples showing high or low PLPP4 expression in lung carcinoma tissues with tumor volume. **g** Percentages and number of samples showing high or low PLPP4 expression in lung carcinoma tissues with different stages. **h** and **i** Expression levels of PLPP4 in lung carcinoma tissues with different stages from lung carcinoma datasets from TCGA and ArrayExpress

Immunohistochemical analysis of PLPP4 expression in 265 non-small cell lung cancer tissues (Additional file 5: Table S1) was further examined. As shown in Fig. 2a and b, PLPP4 expression was primarily detected in the cytoplasm and plasma membrane and the staining intensity of PLPP4 was increased in ADC tissues compared with that in lung granuloma tissues and was further increased in SQC tissues. Furthermore, high expression of PLPP4 was observed in 85/187 ADC tissues (45.5%) and 34/57 SQC tissues (59.6%) (Fig. 2c). Interestingly, we found that the percentage of tissues with high expression of PLPP4 was slightly higher in SQC tissues than that in ADC tissues (Fig. 2c). Statistical analysis of PLPP4 expression and the clinicopathological features of lung carcinoma patients showed that PLPP4 expression positively correlated with the pathological grade, T category and stage of lung carcinoma and the percentage of tissues with high expression of PLPP4 increased gradually with the advance of pathological grade, T category and stage of lung carcinoma (Fig. 2d-g and Additional file 7: Table S7). Analysis of PLPP4 expression in the lung carcinoma datasets from TCGA and ArrayExpress revealed that PLPP4 expression significantly correlated with the stage of lung carcinoma between stage I and stages II-IV (Fig. 2h and i), suggesting that high expression of PLPP4 may be implicated the malignant phenotypes of lung carcinoma. Therefore, our findings indicated that high expression of PLPP4 is positively associated with advanced clinicopathological features in lung carcinoma patients.

### High expression of PLPP4 correlates with poor prognosis in lung carcinoma patients

**Fig. 3:**
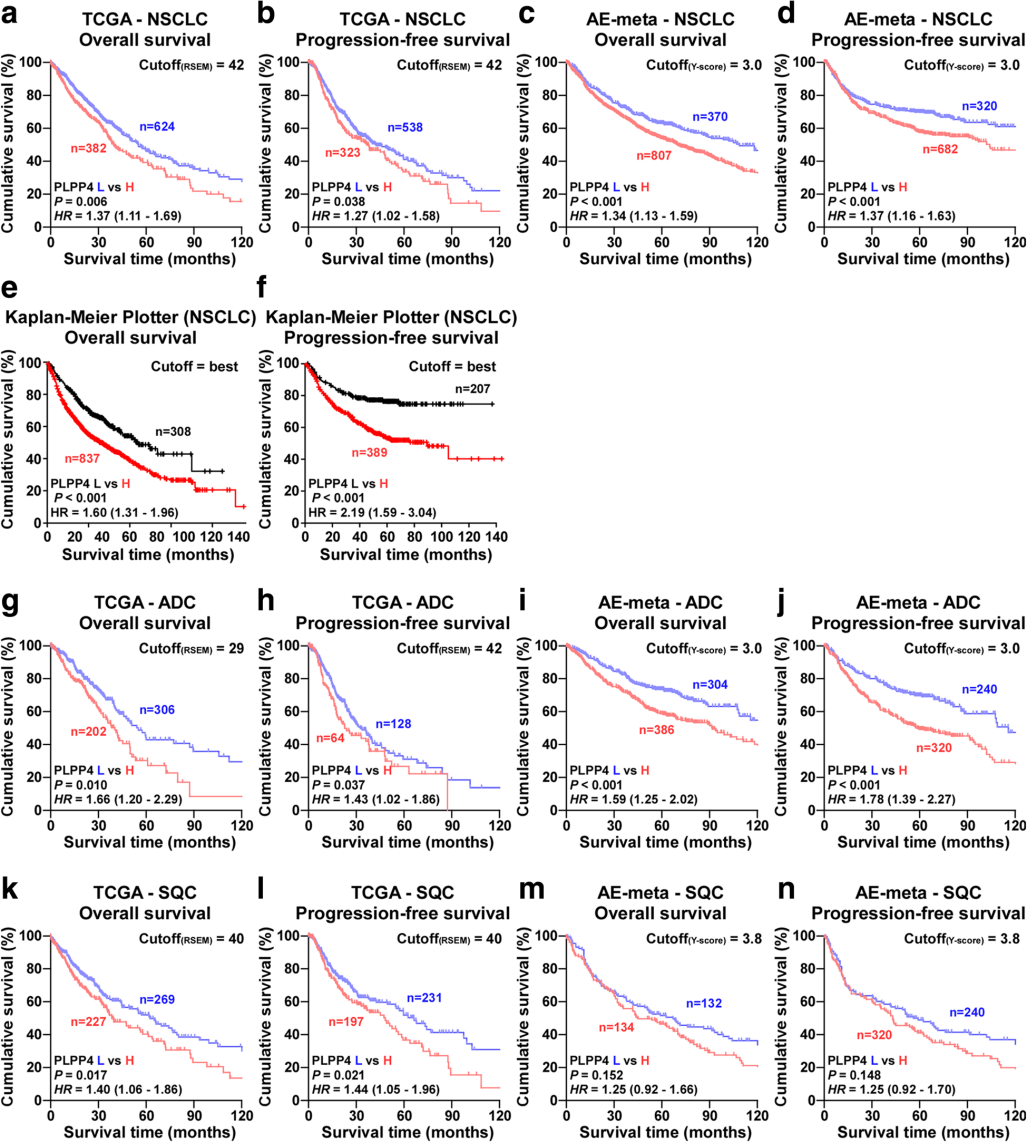
High PLPP4 expression correlates with poor survival in lung carcinoma patients. **a-f** Overall survival and progression-free survival curves from TCGA, ArrayExpress and Kaplan-Meier Plotter profiles for non-small-cell lung carcinoma (NSCLC) patients stratified by high and low expression of PLPP4. **g-j** Overall survival and progression-free survival curves from TCGA and ArrayExpress profiles for ADC patients stratified by high and low expression of PLPP4. **k-n** Overall survival and progression-free survival curves from TCGA and ArrayExpress profiles for SQC patients stratified by high and low expression of PLPP4

To investigate the clinical correlation of PLPP4 with survival in lung carcinoma patients, lung carcinoma datasets from TCGA, ArrayExpress and Kaplan-Meier Plotter were further analyzed and the results revealed that non-small-cell lung carcinoma (NSCLC) patients with high expression of PLPP4 exhibited shorter overall and progression-free survival rates compared with NSCLC patients with low expression of PLPP4 (Fig. 3a-f). In-depth analysis of lung carcinoma TCGA and ArrayExpress datasets showed that ADC or SQC patients with high expression of PLPP4 displayed poor overall and progression-free survival rates (Fig. 3g-l), but not for the SQC dataset from ArrayExpress (Fig. 3m and n). Collectively, these results from publicly available lung carcinoma datasets suggest that the overexpression of PLPP4 correlates with poor prognosis and progression status in lung carcinoma patients.

### Silencing PLPP4 abrogates the proliferation ability of lung carcinoma cells

**Fig. 4:**
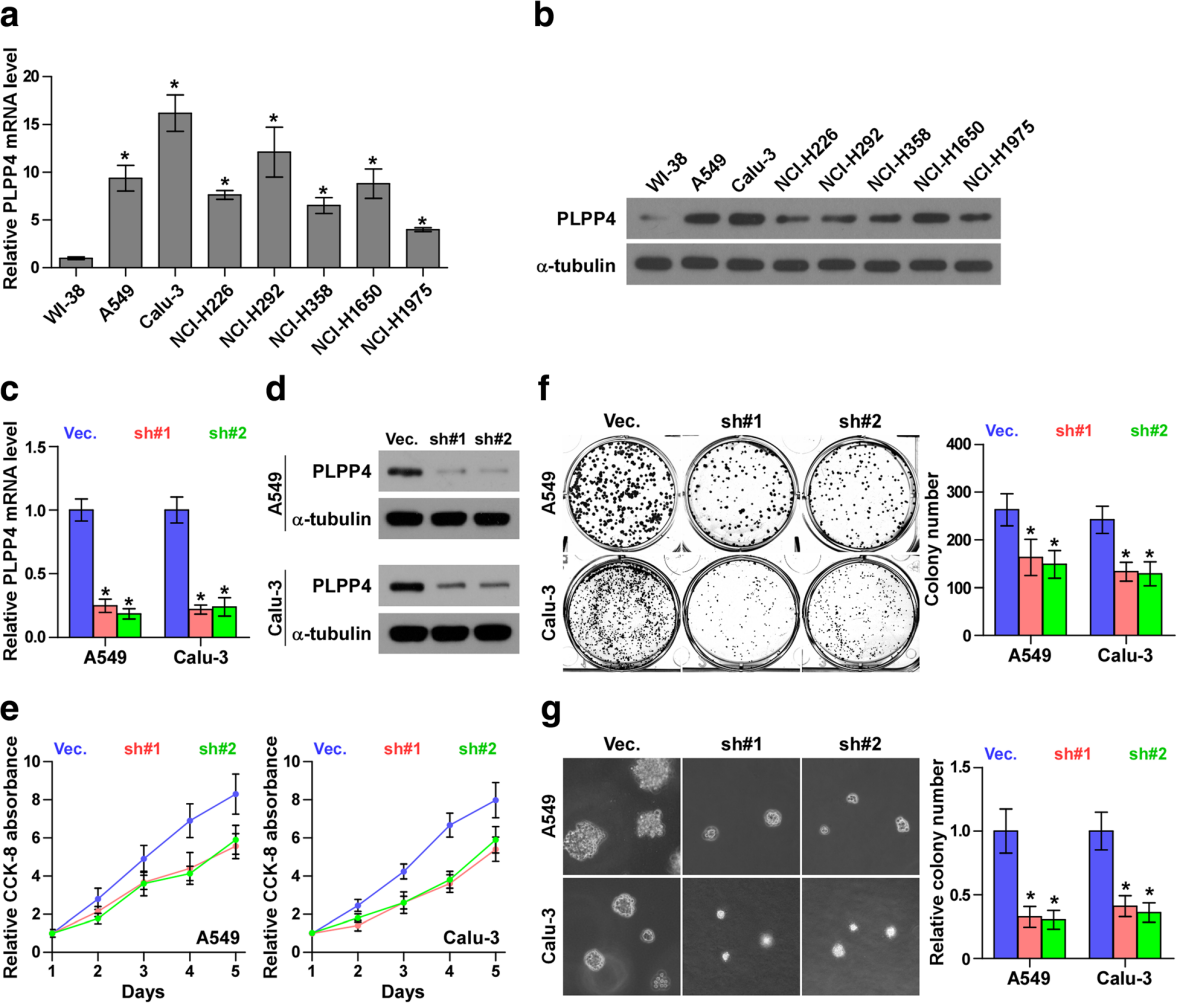
Silencing PLPP4 inhibits proliferation in lung carcinoma cells. **a** and **b** Real-time PCR and Western blotting analysis of PLPP4 expression in WI-38 and lung carcinoma cell lines. GAPDH was used as the endogenous control for RT-PCR and α-Tubulin was used as the loading control in the Western blot. Each bar represents the mean values ± SD of three independent experiments. *P < 0.05. **c** and **d** Real-time PCR and Western blot of the indicated lung carcinoma cells transfected with PLPP4-RNAi-vector, PLPP4-RNAi#1 and PLPP4-RNAi#2. GAPDH was used as the endogenous control for RT-PCR and α-Tubulin was used as the loading control in the Western blot. Each bar represents the mean values ± SD of three independent experiments. *P < 0.05. **e** CCK8 assays revealed that silencing PLPP4 reduced cell viability in lung carcinoma cells. Each bar represents the mean values ± SD of three independent experiments. *P < 0.05. **f** Silencing PLPP4 reduced the mean colony number according to the colony formation assay. Each bar represents the mean values ± SD of three independent experiments. *P < 0.05. **g** Representative micrographs and colony numbers from the anchorage-independent growth assay. Each bar represents the mean values ± SD of three independent experiments. *P < 0.05

To explore the biological roles of PLPP4 in lung carcinoma, we first examined PLPP4 expression levels in the normal lung epithelial cell line WI-38 and seven lung carcinoma cell lines by real-time PCR and Western blotting. As shown in Fig. 4a and b, the mRNA and protein levels of PLPP4 were differentially increased in lung carcinoma cells compared with those in the WI-38 cells. As the highest expression of PLPP4 was in A549 and Calu-3 cells, we constructed PLPP4-stably suppressing A549 and Calu-3 lung carcinoma cells by endogenously knocking down PLPP4 via retroviral infection (Fig. 4c and d). CCK-8 assays were carried out, and the results showed downregulation of PLPP4 decreased viability in lung carcinoma cells (Fig. 4e). Colony formation assays revealed that silencing PLPP4 dramatically inhibited the colony-forming ability of A549 and Calu-3 cells (Fig. 4f). We further performed anchorage-independent growth assays to investigate the effects of PLPP4 on the tumorigenic activity of lung carcinoma cells and found that silencing PLPP4 not only reduced the number of colonies formed by A549 and Calu-3 cells, but also significantly decreased the colony size of lung carcinoma cells (Fig. 4g). Taken together, these findings demonstrated that silencing PLPP4 inhibits the proliferation ability of lung carcinoma cells.

### Silencing PLPP4 retards the G1/S phase transition in lung carcinoma cells

**Fig. 5:**
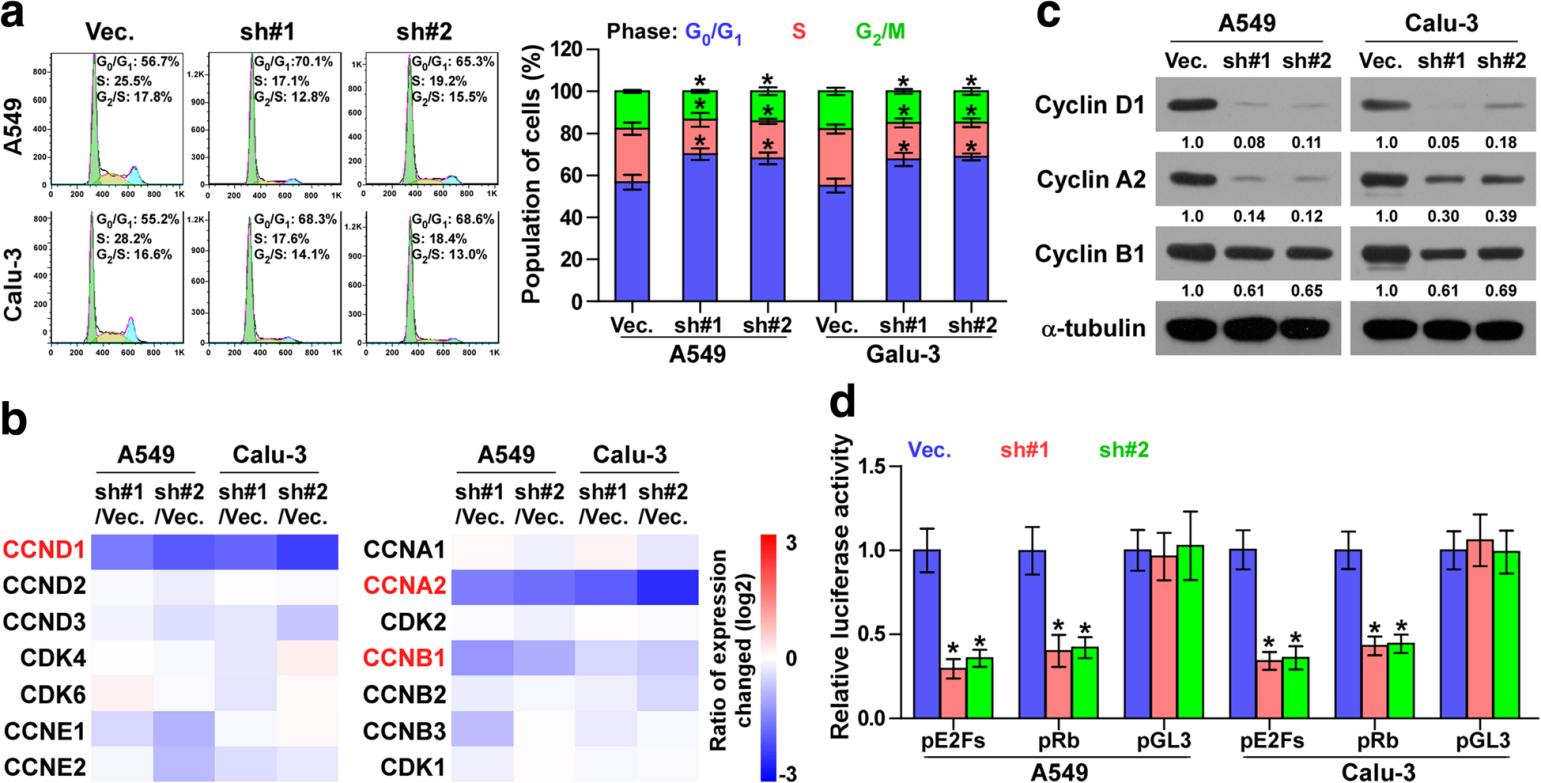
Silencing PLPP4 inhibited cell cycle progression in lung carcinoma cells. **a** Flow cytometric analysis of the indicated lungcarcinoma cells. Each bar represents the mean values ± SD of three independent experiments. *P < 0.05. **b** Real-time PCR analysis of multiple cell cycle regulators in the indicated cells. Transcript levels were normalized by GAPDH expression. **c** Western blot analysis of multiple cell cycle regulators expression in the indicated cells. α-Tubulin was used as the loading control. Protein expression levels of cyclin D1, A2 and B1 were quantified by densitometry using Image J Software, and normalized to the corresponding expression levels of α-tubulin. Sample 1 was used as the standard**. d** Relative E2F and Rb reporter activity in the indicated cells. Each bar represents the mean values ± SD of three independent experiments. *P < 0.05

We then investigated the mechanism underlying the inhibitory effects of PLPP4 downregulation on proliferation in lung carcinoma cells. Flow cytometry analysis showed that silencing PLPP4 dramatically decreased the percentage of cells in the S phase and increased that of cells in the G1/G0 phase, indicating that silencing PLPP4 induced G1/S arrest in lung carcinoma cells (Fig. 5a). Real-time PCR and Western blot analysis revealed that silencing PLPP4 repressed the mRNA and protein expression levels of critical cell cycle regulators of the G_1_/S checkpoint CCND1, CCNA2 and CCNB1 in lung carcinoma cells (Fig. 5b and c). Furthermore, it has been well documented that progression of the cell cycle was promoted by E2F transcription factors. E2F can work with Rb, a negative regulator of the cell cycle, in regulating the G1/S checkpoint. [16, 17]. Luciferase reporter analysis showed that the transcriptional activity of E2F and Rb was dramatically repressed by PLPP4 downregulation; however, the control pGL3-luciferase reporter was not affected by silencing PLPP4 (Fig. 5d). Collectively, these results indicated that downregulation of PLPP4 inhibits proliferation in lung carcinoma cells via eliciting cell cycle arrest.

### Silencing PLPP4 inhibits tumorigenesis in lung carcinoma cells

**Fig. 6:**
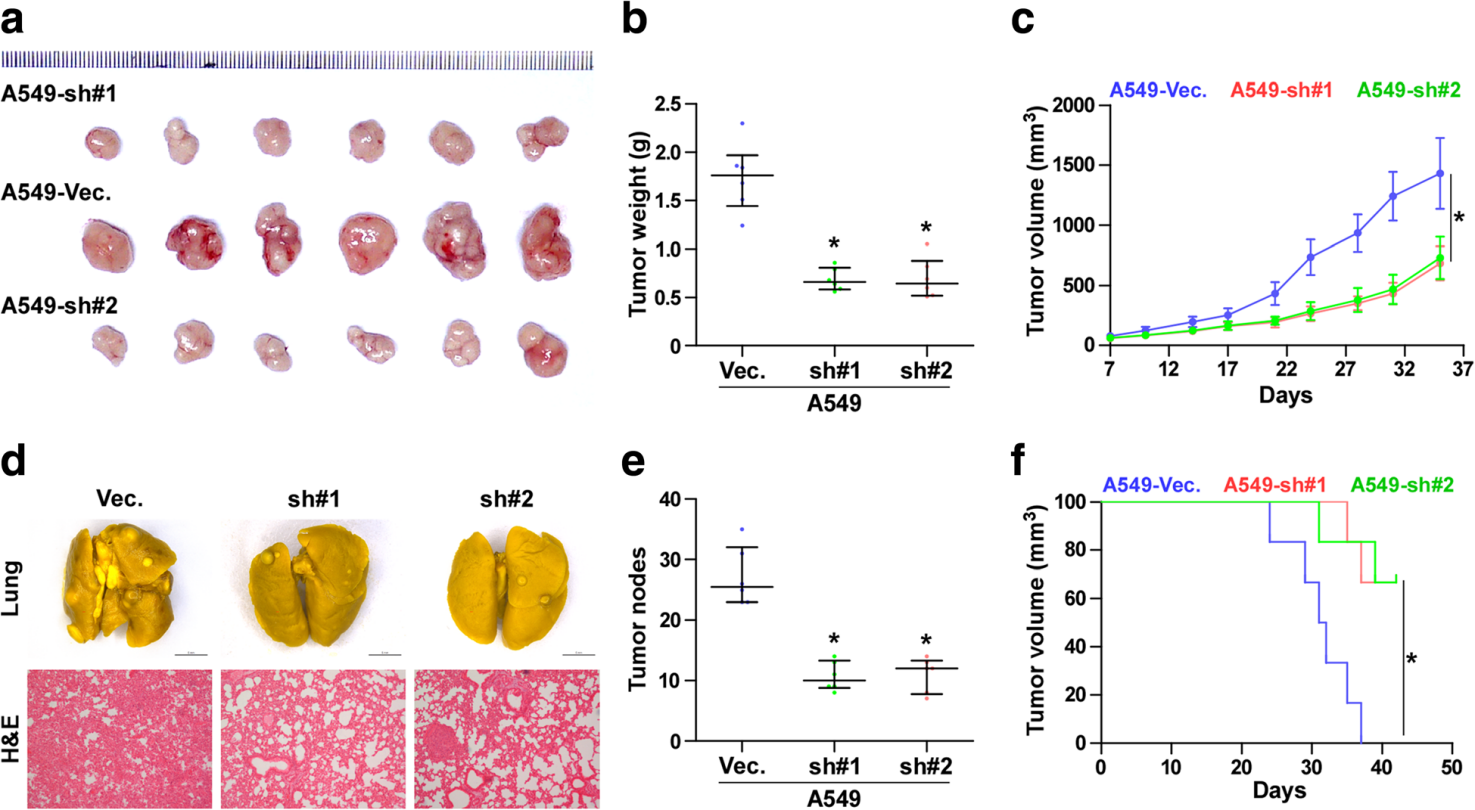
PLPP4 silencing inhibits the tumorigenesis and lung colonization of lung carcinoma cells in vivo. **a** Images of excised tumors from six BALB/c mice at 30 days after injection with the indicated cells. **b** Average weight of excised tumors from the indicated mice. Each bar represents the median values ± quartile values. *P < 0.05. **c** Tumor volumes were measured every five days. Each bar represents the median values ± quartile values. **d** and **e** In vivo lung metastasis assays of A549 cells with PLPP4 knockdown. Lung metastases in mice were confirmed by H&E staining. The number of lung tumor nests in each group was counted under a low power field (LPF) and is presented as the median values ± quartile values (right panel). *P < 0.05. **f** Kaplan-Meier survival curves of the indicated mice

To evaluate the effect of silencing PLPP4 on tumorigenesis in vivo, mice were randomly divided into 3 groups (*n* = 6 per group) and the vector, PLPP4 RNAi#1 and PLPP4 RNAi#2 A549 cells were inoculated subcutaneously into the inguinal folds of the nude mice respectively. As shown in Fig. 6a-f, the tumors formed by the PLPP4-silenced cells were smaller and had decreased tumor volumes and weights compared to those in the control group. We further examined the effects of PLPP4 silencing on the growth ability of A549 cells in the lung. PLPP4silenced and vector-transduced A549 cells were injected into lateral tail veins of the mice, and the growth ability was examined by H&E staining of tumor sections from the lung. We found that downregulating PLPP4 significantly reduced the tumorigenesis of A549 cells in lung and increased the survival in mice (Fig. 6d-f). Collectively, these findings indicate that PLPP4 silencing inhibits the tumorigenesis and lung colonization capabilities of lung carcinoma cells.

### Silencing PLPP4 inhibits Ca^2+^-permeable cationic channel in lung carcinoma cells

**Fig. 7:**
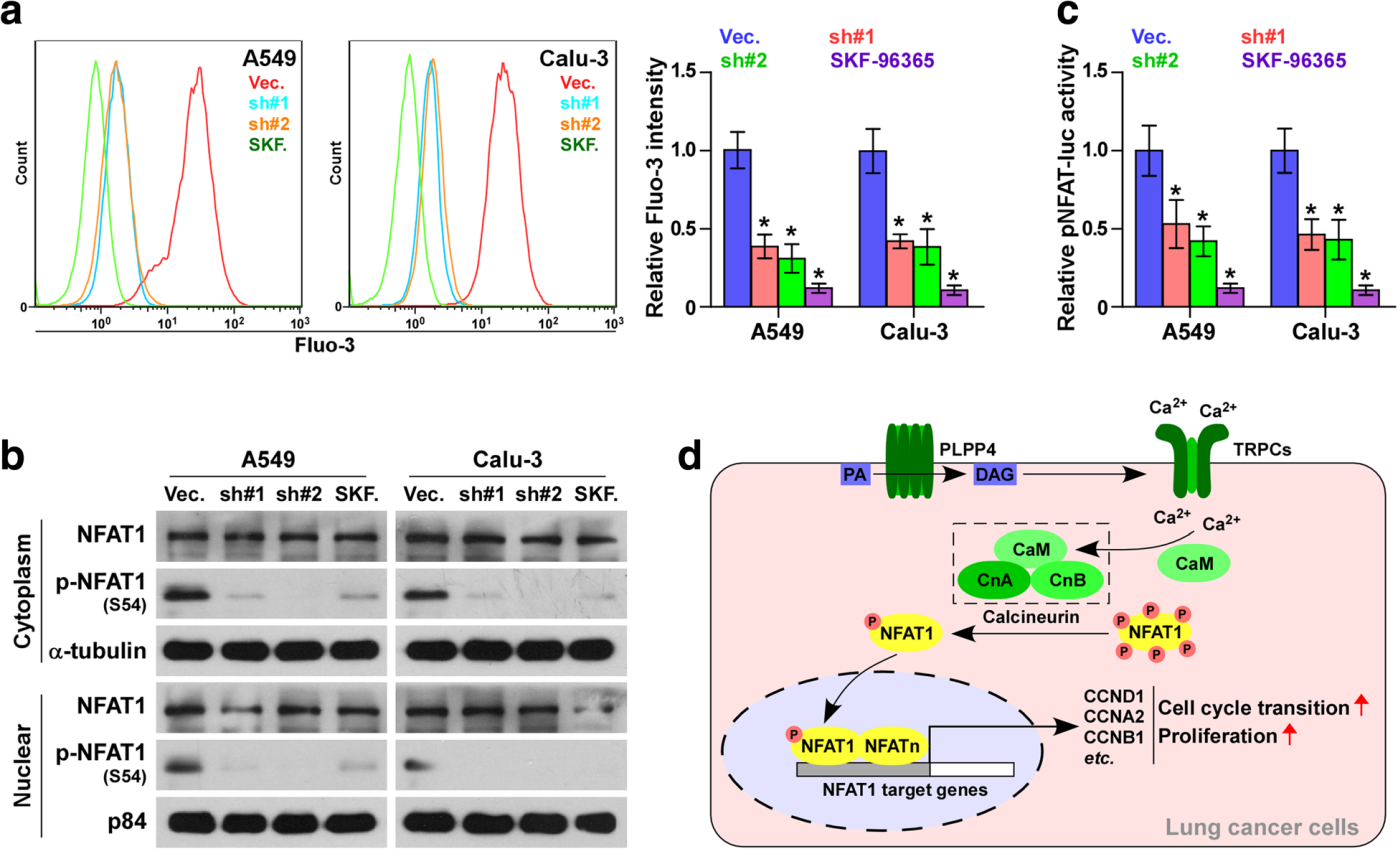
Silencing PLPP4 inhibits Ca^2+^-permeable cationic channel in lung carcinoma cells. **a** FACS analysis showed that silencing PLPP4 decreased intracellular Ca^2+^ in the indicated cells. Each bar represents the mean values ± SD of three independent experiments. *P < 0.05. **b** Western blot analysis of cytoplasmic and nuclear expression of the S54 phosphorylated NFAT1 in the indicated cells. α-Tubulin was used as the loading control and the nuclear protein p84 was used as the nuclear protein marker. **c** Relative NFAT reporter activity in the indicated cells. Each bar represents the mean values ± SD of three independent experiments. *P < 0.05. **d** Hypothetical model illustrating that activation of Ca^2+^-permeable cationic channel by PLPP4 contributes to proliferation and cell cycle progression in lung cancer cells

Several lines of evidence have shown that LPPs catalyze phosphatidic acid, yielding abundant diacylglycerol which further activates the Ca^2+^-permeable cationic channels, leading to the release and influx of Ca^2+^ [3, 5], therefore we further examined the effects of PLPP4 on intracellular Ca^2+^. Fluorescence-activated cell sorting (FACS) analysis showed that silencing PLPP4 decreased intracellular Ca^2+^ in lung carcinoma cells, which was more obvious in cells treated with SKF 96365, a novel inhibitor of receptor-mediated Ca^2+^ entry (5 μm, 24 h) (Fig. 7a). Western blot analysis showed that silencing PLPP4 or SKF 96365 reduced the cytoplasmic S54 phosphorylated NFAT1 and its nuclear translocation (Fig. 7b). Luciferase assays revealed that downregulating PLPP4 or SKF 96365 repressed the transcriptional activity of NFAT in lung carcinoma cells (Fig. 7c). Collectively, our results suggest that silencing PLPP4 inhibits Ca2^+^-permeable cationic channel in lung carcinoma cells (Fig. 7d).

## Discussion

The key findings of the current study show that PLPP4 is dramatically upregulated in lung carcinoma tissues and cells and positively correlates with advanced clinicopathological features, including pathological grade, T category and lung carcinoma stage, and poor prognosis in lung carcinoma patients. Moreover, silencing PLPP4 inhibits the proliferation, cell cycle progression, tumorigenicity and lung metastasis abilities of lung carcinoma cells both in vitro and in vivo. Our findings further reveal that downregulating PLPP4 inhibits Ca2 + −permeable cationic channel in lung carcinoma cells. Therefore, our results elucidate the oncogenic role of PLPP4 in lung carcinoma via elevating intracellular Ca^2+^.

Numerous studies have reported that altered expression of LPPs has been involved in the development and progression of several human cancers. Flanagan et al. reported that downregulating PLPP2 transcriptionally repressed by p53 impaired anchorage-dependent growth of cancer cell lines MCF7, SK-LMS1, MG63, and U2OS, as well as decreased cell proliferation by delaying entry into the S phase of the cell cycle [18]. In addition, it has been demonstrated that PLPP3 was downregulated in approximately 70% of oral squamous cell carcinoma (OSCC) patients, and low expression of PLPP3 positively correlated with TNM stage in OSCC patients [7]. These studies indicated that although belonging to a phosphatidate phosphatase family, different members of the PLPP family function as an oncogene or tumor suppressor in different tumor types. In the current study, we first revealed that PLPP4 was elevated in lung carcinoma tissues. High expression of PLPP4 significantly correlated with advanced clinicopathological features, and poor overall and progression-free survival in lung carcinoma patients. In vitro and in vivo assays demonstrated that silencing PLPP4 inhibited the proliferation and cell cycle progression, and the tumorigenesis of lung carcinoma cells in subcutaneous, as well as in the lung. Thus, our results indicate that high expression of PLPP4 is implicated in the progression of lung carcinoma via promoting the proliferation and cell cycle in lung carcinoma cells.

PLPP4 was first identified by DNA microarray, cDNA dot blot analysis, and reverse transcription-PCR techniques in paired breast cancer tissues [19]. One year later, Takeuchi et al. found several unknown ESTs via oligonucleotide microarray analysis and cloned two novel cDNAs encoding the putative 264-residue protein PLPP5 and the 271-residue protein PLPP4 [20]. Furthermore, Manzano and colleagues reported that PLPP4 was upregulated in only the estrogen receptor (ER)-ERBB2+ subgroup, but not in other subtypes of breast cancer, suggesting a specific role of PLPP4 in ER-ERBB2+ subtypes [21]. However, the clinical significance and biological role of PLPP4 in cancers remain blank. In this study, through analyzing the expression levels of LPPs proteins in the lung carcinoma RNA expression profile datasets from TCGA, we first found that PLPP4 was considerably elevated compared with other LPPs in lung carcinoma tissues. Overexpression of PLPP4 was further observed in our lung carcinoma tissues and cells and positively correlated with advanced clinicopathological features and poor prognosis. Moreover, silencing PLPP4 repressed the proliferation, tumorigenicity and lung metastasis abilities of lung carcinoma cells both in in vitro and in vivo, as well as inhibited Ca^2+^-permeable cationic channel in lung carcinoma cells. Therefore, our findings indicate that PLPP4 plays an important role in lung carcinoma.

Numerous studies have demonstrated that LPPs catalyze the conversion of phosphatidic acid generated from phosphatidylcholine to diacylglycerol [3, 4]. The increment of diacylglycerol concentration could directly activate some transient receptor potential channel (TRPC) family members without depleting intracellular Ca^2+^ stores, leading to the release and influx of intracellular Ca^2+^ [22, 23]. As a universal second messenger, calcium is involved in several important cellular processes, including growth, differentiation, and programmed cell death [24, 25], as well as in pathological diseases, such as cancer development [5, 26]. Therefore, these studies suggested that LPPs may contribute to the development of cancer via diacylglycerol-gated Ca^2+^-permeable cationic channel [4, 5]. Furthermore, therapies aiming at inhibiting intracellular Ca^2+^ may be favorable for the treatment of cancer [27, 28]. In the current study, our results found that silencing PLPP4 effectively reduced intracellular Ca^2+^ and S54 phosphorylated levels of NFAT1, which further inhibited the proliferation and tumorigenesis in lung carcinoma cells. Our findings indicate that inhibition of PLPP4 may be used as a novel therapeutic strategy in the treatment of lung carcinoma.

It has been widely documented that LPPs may serve as a potential marker for the diagnosis of cancer or as a putative therapeutic target in the treatment of cancers. Amin and colleagues have shown that PLPP1, a downstream ErbB2 signaling target, may be used as a diagnostic marker in breast cancer [29]. Moreover, it was reported that a series of in vitro data, including anchorage-dependent growth and proliferation assays, validated PLPP2 as a putative therapeutic target for treatment of cancer [18]. In this study, our results implied that high expression of PLPP4 was observed in lung adenocarcinoma and lung squamous cell carcinoma tissues and was positively associated with pathological grade, T category and lung carcinoma stage in patients, suggesting that PLPP4 holds promise as a novel marker for the diagnosis of lung carcinoma. Furthermore, subcutaneous and lung colonization models showed that downregulating PLPP4 dramatically inhibited the tumorigenesis of lung carcinoma cells in a mouse model, indicating that PLPP4 may hold potential applicable value as a therapeutic target against lung carcinoma. However, further validation needs to be warrant in a large series of studies.

## Conclusion

In summary, our results demonstrate that high expression of PLPP4 promotes the proliferation and tumorigenesis of lung carcinoma cells, as well as enhances Ca^2+^-permeable cationic channel, suggesting that PLPP4-mediated elevation of intracellular Ca^2+^ contributes to lung carcinoma cell proliferation by promoting cell cycle progression. Therefore, our data may lead us to further in vivo studies, namely, inhibition of PLPP4 may be used as a complementary strategy in the treatment of lung carcinoma.

## Additional files

Additional file 1: Table S1. The basic information of 8 lung cancer patients for PLPP4 mRNA and protein expression analysis. (PDF 51 kb)

Additional file 2: Table S2. The basic information of 43 patients with benign pulmonary lesions for PLPP4 immunohistochemical staining analysis. (PDF 51 kb)

Additional file 3: Table S3. The basic information of 265 patients with non-small cell lung cancer for PLPP4 immunohistochemical staining analysis. (PDF 58 kb)

Additional file 4: Table S4. A list of primers used in the reactions for real-time RT-PCR. (PDF 14 kb)

Additional file 5: Table S5. A list of primers used in the reactions for clone PCR. (PDF 5 kb)

Additional file 6: Table S6. A list of high throughput datasets used by YuGene meta-analysis for PLPP4 mRNA expression. (PDF 22 kb)

Additional file 7: Table S7. The relationship between PLPP4 IHC expression level and clinical pathological characteristics in 265 patients with non-small cell lung cancer. (PDF 59 kb)

## Abbreviations

ADC: Adenocarcinoma
H&E: Hematoxylin and Eosin Stain
IHC: Immunohistochemistry
LPPs: Lipid phosphate phosphatases
NFAT1: Nuclear Factor Of Activated T-Cells 2
PCR: Polymerase Chain Reaction
PLPP4: Phospholipid Phosphatase 4
Rb: RB Transcriptional Corepressor 1
SQC: squamous cell carcinoma
TCGA: The Cancer Genome Atlas
TCGA: The Cancer Genome Atlas
