# Supplementary material for: Phospholipid Phosphatase 4 promotes proliferation and tumorigenesis, and activates Ca2+-permeable Cationic Channel in lung carcinoma cells
Source: Mol Cancer. 2017 Aug 29;16:147. doi: 10.1186/s12943-017-0717-5 (PMC5576330; doi:10.1186/s12943-017-0717-5)
Supplement: Supplementary file 1 — The basic information of 8 lung cancer patients for PLPP4 mRNA and protein expression analysis. (PDF 51 kb) [file 12943_2017_717_MOESM1_ESM.pdf]

Table S1. The basic information of 8 lung cancer patients for PLPP4 mRNA and protein expression analysis.

		Cases (n)	Percentage (%)
Histologic	ADC	5	62.5
	SQC	2	25.0
	ASC	1	12.5
Gender	Male	5	62.5
	Female	3	37.5
Age	<60	5	62.5
	≥60	3	37.5
Grade (exclude: ASC)	G1	2	28.6
	G2	3	42.8
	G3	2	28.6
Stage	Stage I	3	37.5
	Stage II	3	37.5
	Stage III	2	25.0
	Stage IV	0	0.0

* ADC: Adenocarcinoma; SQC: Squamous carcinoma; ASC: Adenosquamous carcinoma.
