# Supplementary material for: Phospholipid Phosphatase 4 promotes proliferation and tumorigenesis, and activates Ca2+-permeable Cationic Channel in lung carcinoma cells
Source: Mol Cancer. 2017 Aug 29;16:147. doi: 10.1186/s12943-017-0717-5 (PMC5576330; doi:10.1186/s12943-017-0717-5)
Supplement: Supplementary file 2 — The basic information of 43 patients with benign pulmonary lesions for PLPP4 immunohistochemical staining analysis. (PDF 51 kb) [file 12943_2017_717_MOESM2_ESM.pdf]

Table S2. The basic information of 43 patients with benign pulmonary lesions for PLPP4 immunohistochemical staining analysis.

		Cases (n)	Percentage (%)
Gender	Male	33	76.7
	Female	10	23.3
Age	<60	31	72.1
	≥60	9	20.9
	N/A	3	7.0
Type of diseases	Amyloidosis	1	2.3
	Fungal infection	2	4.7
	Hamartoma	3	7.0
	Hyperplasia	19	44.2
	Metaplasia	1	2.3
	Pneumonia	5	11.6
	Pulmonary bulla	5	11.6
	Sclerosing hemangioma	1	2.3
	Tuberculosis	6	14.0

* N/A: Not available.
