# Supplementary material for: Phospholipid Phosphatase 4 promotes proliferation and tumorigenesis, and activates Ca2+-permeable Cationic Channel in lung carcinoma cells
Source: Mol Cancer. 2017 Aug 29;16:147. doi: 10.1186/s12943-017-0717-5 (PMC5576330; doi:10.1186/s12943-017-0717-5)
Supplement: Supplementary file 3 — The basic information of 265 patients with non-small cell lung cancer for PLPP4 immunohistochemical staining analysis. (PDF 58 kb) [file 12943_2017_717_MOESM3_ESM.pdf]

Table S3. The basic information of 265 patients with non-small cell lung cancer for PLPP4 immunohistochemical staining analysis.

		Cases (n)	Percentage (%)
Histologic	ADC	187	70.6
	SQC	57	21.5
	Other	21	7.9
Gender	Male	175	66.0
	Female	90	34.0
Age	<60	100	37.7
	≥60	164	61.9
	N/A	1	0.4
Grade	G1	24	9.8
(excluded other)	G2	114	46.7
	G3	93	38.1
	N/A	13	5.3
T classification	T1	78	29.4
	T2	123	46.4
	T3	39	14.7
	T4	14	5.3
	N/A	11	4.1
N classification	N0	158	59.6
	N1	63	23.8
	N2	28	10.6
	N3	2	0.8
	N/A	14	5.3
M classification	M0	232	87.5
	M1	14	5.3
	N/A	19	7.2

Stage	Stage I	93	35.1
	Stage II	89	33.6
	Stage III	48	18.1
	Stage IV	14	5.3
	N/A	21	7.9

* ADC: Adenocarcinoma; SQC: Squamous carcinoma; N/A: Not available.

** Other histologic cancers included: Adenosquamous carcinoma (ASC), Large cell neuroendocrine carcinoma (LCNE), Undifferentiated carcinoma (UDC), Epidermoid carcinoma (EPC), Sarcomatoid carcinoma (SARC), and Pleomorphic carcinoma (PMC).
