# Supplementary material for: Phospholipid Phosphatase 4 promotes proliferation and tumorigenesis, and activates Ca2+-permeable Cationic Channel in lung carcinoma cells
Source: Mol Cancer. 2017 Aug 29;16:147. doi: 10.1186/s12943-017-0717-5 (PMC5576330; doi:10.1186/s12943-017-0717-5)
Supplement: Supplementary file 4 — A list of primers used in the reactions for real-time RT-PCR. (PDF 14 kb) [file 12943_2017_717_MOESM4_ESM.pdf]

Table S4. A list of primers used in the reactions for real-time RT-PCR.

Gene name		Sequence
PLPP4	forward	5'-TTTGGATCCGTTCCAGAGAG-3'
	reverse	5'-CAGGGGTGTGAGGAAAGAAA-3'
CCND1	forward	5'-GCCCTCGGTGTCCTACTTC-3'
	reverse	5'-CTCCTCCTCGCACTTCTGTT-3'
CCND2	forward	5'-GGTCGGGTTTTCAATCACAC-3'
	reverse	5'-CCTCTTCACCTCCCTTCAACT-3'
CCND3	forward	5'-TCCTCTCCCATTGTCCCTCT-3'
	reverse	5'-CCACCAGCCTAAACCTTGC-3'
CDK4	forward	5'-CAGCTACCAGATGGCACTTACA-3'
	reverse	5'-CAAAGATACAGCCAACACTCCA-3'
CDK6	forward	5'-GTCAGGTTGTTTGATGTGTGC-3'
	reverse	5'-CGGTGTGAATGAAGAAAGTCC-3'
CCNE1	forward	5'-CGGTATATGGCGACACAAGA-3'
	reverse	5'-ACATACGCAAACTGGTGCAA-3'
CCNE2	forward	5'-AGGAAAACTACCCAGGATGTCA-3'
	reverse	5'-ATCAGGCAAAGGTGAAGGATTA-3'
CCNA1	forward	5'-GGAAGGCATTTTCTGATCCA-3'
	reverse	5'-GCTAGGGCTGCTAACTGCAA-3'
CCNA2	forward	5'-ATGTCACCGTTCCTCCTTG-3'
	reverse	5'-GGGCATCTTCACGCTCTATT-3'
CDK2	forward	5'-TGCCTGATTACAAGCCAAGTT-3'
	reverse	5'-GAGTCGAAGATGGGGTACTGG-3'
CCNB1	forward	5'-TGAGGAAGAGCAAGCAGTCA-3'
	reverse	5'-ATGGTCTCCTGCAACAACCT-3'
CCNB2	forward	5'-ACTGCTCTGCTCTTGGCTTC-3'
	reverse	5'-TTTCTCGGATTTGGGAACTG-3'
CCNB3	forward	5'-AGATCCACCAGCTTCACTGC-3'
	reverse	5'-GTGACATGAGGGCCATTCTT-3'
CDK1	forward	5'-GGTCAAGTGGTAGCCATGAAA-3'
	reverse	5'-CCAGGAGGGATAGAATCCAAG-3'
GAPDH	forward	5'-GCACCGTCAAGGCTGAGAAC-3'
	reverse	5'-TGGTGAAGACGCCAGTGGA-3'
