# Supplementary material for: Phospholipid Phosphatase 4 promotes proliferation and tumorigenesis, and activates Ca2+-permeable Cationic Channel in lung carcinoma cells
Source: Mol Cancer. 2017 Aug 29;16:147. doi: 10.1186/s12943-017-0717-5 (PMC5576330; doi:10.1186/s12943-017-0717-5)
Supplement: Supplementary file 5 — A list of primers used in the reactions for clone PCR. (PDF 5 kb) [file 12943_2017_717_MOESM5_ESM.pdf]

Table S5. A list of primers used in the reactions for clone PCR.

Gene name		Sequence
shPLPP4#1	forward	5'-CCGGCCC-GGAGTGATGAACTCGGAAATG -TTCAAGAGA-CATTTCCGAGTTCATCACTCC -TTTTTG-3'
	reverse	5'-AATTCAAAAA-GGAGTGATGAACTCGGAAATG -TCTCTTGAA-CATTTCCGAGTTCATCACTCC -GGG-3'
shPLPP4#2	forward	5'-CCGGCCC-GCTTCACGACGTTCTACTTGG -TTCAAGAGA-CCAAGTAGAACGACGTGAAGC -TTTTTA-3'
	reverse	5'-AATTCAAAAA-GCTTCACGACGTTCTACTTGG -TCTCTTGAA-CCAAGTAGAACGACGTGAAGC -GGG-3'
