# Supplementary material for: Phospholipid Phosphatase 4 promotes proliferation and tumorigenesis, and activates Ca2+-permeable Cationic Channel in lung carcinoma cells
Source: Mol Cancer. 2017 Aug 29;16:147. doi: 10.1186/s12943-017-0717-5 (PMC5576330; doi:10.1186/s12943-017-0717-5)
Supplement: Supplementary file 6 — A list of high throughput datasets used by YuGene meta-analysis for PLPP4 mRNA expression. (PDF 22 kb) [file 12943_2017_717_MOESM6_ESM.pdf]

Table S6. A list of high throughput datasets used by YuGene meta-analysis for PLPP4 mRNA expression.

Series	Tiles (Citation)	Samples	Region
E-GEOD-10445	Transcription profiling of human stage IB lung adenocarcinomas and large cell carcinomas to predict outcomes (Merlion lung cancer study)[1]	n = 72	France
E-GEOD-12667	Transcription profiling by array of human lung adenocarcinoma tissue from smokers, ex-smokers and people who have never smoked[2]	n = 75	USA
E-GEOD-18842	Transcription profiling by array of human non-small cell lung cancer tissue[3]	n = 91	Spain
E-GEOD-19188	Transcription profiling by array of human lung cancer samples[4]	n =156	Netherlands
E-GEOD-28571	Expression data of different histological subgroups of non-small cell lung cancer in two patient populations with different survival outcomes[5]	n =100	Sweden
E-GEOD-30219	Gene expression in lung cancer identifies a group of metastatic-prone tumors[6]	n =307	France
E-GEOD-31210	Gene expression data for pathological stage I-II lung adenocarcinomas[7]	n =245	Japan
E-GEOD-31908	Primary Lung Cancer Specimens	n =17	USA
E-GEOD-33356	Genome-wide screening of genomic alterations and transcriptional modulation in non-smoking female lung cancer in Taiwan[8]	n =120	Taiwan
E-GEOD-33532	Intratumor heterogeneity of gene expression profiles in early stage non-small cell lung cancer	n =100	Germany
E-GEOD-37745	Biomarker discovery in non-small cell lung cancer: integrating gene expression profiling, meta-analysis and tissue microarray validation[9]	n =196	Sweden
E-GEOD-40791	Usp44 binds centrin to regulate centrosome positioning and suppress tumorigenesis[10]	n =194	USA

E-GEOD-43580	SBV - Gene Expression Profiles of Lung Cancer Tumors - Adenocarcinomas and Squamous Cell Carcinomas[11]	n =150	Switzerland
E-GEOD-50081	Validation of a histology-independent prognostic gene signature for early stage, non-small cell lung cancer including stage IA patients[12]	n =181	Canada
E-GEOD-77803	Retrospective and prospective validation of a 33-gene signature to predict recurrence of lung cancer after surgery	n =156	Denmark
E-MTAB-923	Transcription profiling by array of human lung samples from patients with adenocarcinoma to identify likely drivers of cell proliferation	n =103	France
E-MTAB-1727	DNA copy number variation profiling and transcription profiling by array of 93 human lung squamous cell carcinoma samples[13]	n =109	France

References

1. Broet P, Camilleri-Broet S, Zhang S, Alifano M, Bangarusamy D, Battistella M, Wu Y, Tuefferd M, Regnard JF, Lim E *et al*: **Prediction of clinical outcome in multiple lung cancer cohorts by integrative genomics: implications for chemotherapy selection**. *Cancer research* 2009, **69**(3):1055-1062.
2. Ding L, Getz G, Wheeler DA, Mardis ER, McLellan MD, Cibulskis K, Sougnez C, Greulich H, Muzny DM, Morgan MB *et al*: **Somatic mutations affect key pathways in lung adenocarcinoma**. *Nature* 2008, **455**(7216):1069-1075.
3. Sanchez-Palencia A, Gomez-Morales M, Gomez-Capilla JA, Pedraza V, Boyero L, Rosell R, Farez-Vidal ME: **Gene expression profiling reveals novel biomarkers in nonsmall cell lung cancer**. *International journal of cancer* 2011, **129**(2):355-364.
4. Hou J, Aerts J, den Hamer B, van Ijcken W, den Bakker M, Riegman P, van der Leest C, van der Spek P, Foekens JA, Hoogsteden HC *et al*: **Gene expression-based classification of non-small cell lung carcinomas and survival prediction**. *PloS one* 2010, **5**(4):e10312.
5. Micke P, Edlund K, Holmberg L, Kultima HG, Mansouri L, Ekman S, Bergqvist M, Scheibenflug L, Lamberg K, Myrdal G *et al*: **Gene copy number aberrations are associated with survival in histologic subgroups of non-small cell lung cancer**. *J Thorac Oncol* 2011, **6**(11):1833-1840.
6. Rousseaux S, Debernardi A, Jacquiau B, Vitte AL, Vesin A, Nagy-Mignotte H,

Moro-Sibilot D, Brichon PY, Lantuejoul S, Hainaut P *et al*: **Ectopic activation of germline and placental genes identifies aggressive metastasis-prone lung cancers**. *Sci Transl Med* 2013, **5**(186):186ra166.

7. Okayama H, Kohno T, Ishii Y, Shimada Y, Shiraishi K, Iwakawa R, Furuta K, Tsuta K, Shibata T, Yamamoto S *et al*: **Identification of genes upregulated in ALK-positive and EGFR/KRAS/ALK-negative lung adenocarcinomas**. *Cancer research* 2012, **72**(1):100-111.
8. Lu TP, Lai LC, Tsai MH, Chen PC, Hsu CP, Lee JM, Hsiao CK, Chuang EY: **Integrated analyses of copy number variations and gene expression in lung adenocarcinoma**. *PloS one* 2011, **6**(9):e24829.
9. Botling J, Edlund K, Lohr M, Hellwig B, Holmberg L, Lambe M, Berglund A, Ekman S, Bergqvist M, Ponten F *et al*: **Biomarker discovery in non-small cell lung cancer: integrating gene expression profiling, meta-analysis, and tissue microarray validation**. *Clinical cancer research : an official journal of the American Association for Cancer Research* 2013, **19**(1):194-204.
10. Zhang Y, Foreman O, Wigle DA, Kosari F, Vasmatzis G, Salisbury JL, van Deursen J, Galardy PJ: **USP44 regulates centrosome positioning to prevent aneuploidy and suppress tumorigenesis**. *The Journal of clinical investigation* 2012, **122**(12):4362-4374.
11. Tarca AL, Lauria M, Unger M, Bilal E, Boue S, Kumar Dey K, Hoeng J, Koeppl H, Martin F, Meyer P *et al*: **Strengths and limitations of microarray-based phenotype prediction: lessons learned from the IMPROVER Diagnostic Signature Challenge**. *Bioinformatics* 2013, **29**(22):2892-2899.
12. Der SD, Sykes J, Pintilie M, Zhu CQ, Strumpf D, Liu N, Jurisica I, Shepherd FA, Tsao MS: **Validation of a histology-independent prognostic gene signature for early-stage, non-small-cell lung cancer including stage IA patients**. *J Thorac Oncol* 2014, **9**(1):59-64.
13. Brambilla C, Laffaire J, Lantuejoul S, Moro-Sibilot D, Mignotte H, Arbib F, Toffart AC, Petel F, Hainaut P, Rousseaux S *et al*: **Lung squamous cell carcinomas with basaloid histology represent a specific molecular entity**. *Clinical cancer research : an official journal of the American Association for Cancer Research* 2014, **20**(22):5777-5786.
