# Supplementary material for: Phospholipid Phosphatase 4 promotes proliferation and tumorigenesis, and activates Ca2+-permeable Cationic Channel in lung carcinoma cells
Source: Mol Cancer. 2017 Aug 29;16:147. doi: 10.1186/s12943-017-0717-5 (PMC5576330; doi:10.1186/s12943-017-0717-5)
Supplement: Supplementary file 7 — The relationship between PLPP4 IHC expression level and clinical pathological characteristics in 265 patients with non-small cell lung cancer. (PDF 59 kb) [file 12943_2017_717_MOESM7_ESM.pdf]

Table S7. The relationship between PLPP4 IHC expression level and clinical pathological characteristics in 265 patients with non-small cell lung cancer.

Parameters	Number of cases	PLPP4 IHC expression		P values
		Low	High	
Histologic		137	128	
ADC	187	102	85	0.061
SQC	57	23	34	
Gender				
Male	175	96	79	0.151
Female	90	41	49	
Age				
<60	100	53	47	0.779
≥60	164	84	80	
Grade				
G1-G2	138	91	47	<0.001*
G3	93	30	63	
T classification				
T1	78	50	28	0.008*
T2-4	176	81	95	
N classification				
N0	158	92	66	0.066
N1-3	93	43	50	
M classification				
M0	232	129	103	0.147
M1	14	5	9	
Stage				
I-II	182	102	80	0.010*
III-IV	62	23	39	

* IHC: Immunohistochemistry; ADC: Adenocarcinoma; SQC: Squamous carcinoma.
